# Synthesizing Conductive Metal–Organic Framework Nanosheets for High-Performing Chemiresistive Sensors

**DOI:** 10.1021/acsami.5c00064

**Published:** 2025-03-15

**Authors:** Chuanhui Huang, Shirong Huang, Wei Wang, Xing Huang, Arezoo Dianat, Rashid Iqbal, Geping Zhang, Naisa Chandrasekhar, Luis Antonio Panes-Ruiz, Yang Lu, Zhongquan Liao, Bergoi Ibarlucea, Chenchen Wang, Xinliang Feng, Gianaurelio Cuniberti, Renhao Dong

**Affiliations:** † Center for Advancing Electronics Dresden (Cfaed) and Faculty of Chemistry and Food Chemistry, Technische Universität Dresden, 01062 Dresden, Germany; ‡ Institute for Materials Science and Max Bergmann Center for Biomaterials, TUD Dresden University of Technology, 01062 Dresden, Germany; § Key Laboratory of Colloid and Interface Chemistry of the Ministry of Education, School of Chemistry and Chemical Engineering, Shandong University, Jinan 250100, China; ∥ Department of Chemistry, The University of Hong Kong, Hong Kong 999077, China; ⊥ Materials Innovation Institute for Life Sciences and Energy (MILES), HKU-SIRI, Shenzhen 518048, China; # Fraunhofer Institute for Ceramic Technologies and Systems (IKTS), Maria-Reiche-Strasse 2, 01109 Dresden, Germany; ∇ Department of Synthetic Materials and Functional Devices, Max Planck Institute for Microstructure Physics, D-06120 Halle (Saale), Germany; ○ Dresden Center for Computational Materials Science (DCMS), TUD Dresden University of Technology, 01062 Dresden, Germany

**Keywords:** conductive metal−organic frameworks, nanosheets, sacrifice template approach, flexibility, chemiresistive sensors

## Abstract

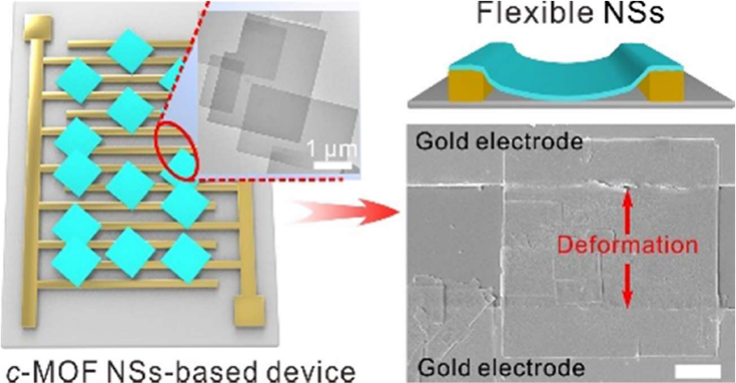

Two-dimensional conjugated metal–organic frameworks (2D *c*-MOFs) are emerging as unique electrode materials with great potential for electronic applications. However, traditional devices based on *c*-MOFs often utilize them directly in the powder or nanoparticle form, leading to weak adhesion to the device substrate and resulting in low stability and high noise levels in the final device. In this study, we present a novel approach utilizing thin *c*-MOFs synthesized via a general MOF nanosheet sacrifice approach, enhancing their aspect ratio and flexibility for high-performance electronic applications. The resultant benzene-based Cu-BHT nanosheets feature a thin thickness (around 5 nm) and a high aspect ratio (>100), affording Cu-BHT exceptional flexibility with a 10-fold decrease in Young’s modulus (0.98 GPa) and hardness (0.09 GPa) compared to bulk Cu-BHT nanoparticles (10.79 and 0.75 GPa, respectively). This heightened flexibility enables the Cu-BHT nanosheets to conform to the channels of the electrodes, ensuring robust adhesion to the electrode substrate and improving device stability. As a proof-of-concept, the chemiresistive nanosensor based on Cu-BHT nanosheets demonstrates an 8.0-fold decrease in the coefficient of variation of the response intensity and a 47.1-fold increase in the signal-to-noise ratio compared to sensors based on bulk Cu-BHT nanoparticles. Combined with the machine learning algorithms, the Cu-BHT nanosensor demonstrates outstanding performance in identifying and discriminating multiple volatile organic compounds at room temperature with an average accuracy of 97.9%, surpassing the thus-far-reported chemiresistive sensors.

## Introduction

Two-dimensional conjugated metal–organic frameworks (2D *c*-MOFs) with in-plane extended π-conjugation possess abundant excellent physicochemical properties, such as intrinsic porosity, diverse structures, electrical conductivity, and tailorable band gaps.^1−4^ Consequently, *c*-MOFs offer significant advantages in various applications, such as energy storage,^5,6^ thermoelectrics,^7,8^ electrocatalysis,^9,10^ superconductors,^11^ gas sensors,^12−14^ etc. For instance, chemiresistive devices based on *c*-MOFs demonstrate superior performance in response intensity, selectivity, and response speed toward target gas molecules.^15−17^ However, in reported devices utilizing *c*-MOFs, integration often relies on drop-casting of *c*-MOF samples in the form of powder or nanoparticles (NPs).^18−20^ Due to the rigid nature of *c*-MOF NPs and their limited contact with the device substrate, detachment frequently occurs, resulting in low stability and high noise in the final device. This presents a significant challenge for the practical application of *c*-MOF-based electronics.

Pioneering efforts have demonstrated that nanosheets (NSs) exhibit excellent physical and mechanical properties, such as ultrathin thickness, remarkable flexibility, stretchability, and superior adhesion to device substrates, and have become one of the optimal materials for devices due to these remarkable characteristics.^21−23^ This implies that producing *c*-MOFs in an NS structure could be an excellent solution for enhancing device stability and signal-to-noise ratio, as flexible *c*-MOF NSs boast exceptional adhesion for intimate device integration. However, the two reported strategies for preparing *c*-MOF NSs, namely, top-down and bottom-up methods, are challenging to deem perfect. For example, ball-milling mechanical exfoliation, considered a viable top-down method, has been introduced to prepare phthalocyanine-based *c*-MOF NSs.^24^ Although this top-down method is straightforward, it is applicable to only a limited range of *c*-MOFs and often leads to fragmentation and morphological damage due to the strong π–π stacking within the *c*-MOF crystals. Recently, our group also developed a bottom-up strategy, specifically a surfactant-assisted solution synthesis, for producing ultrathin *c*-MOF NSs.^25^ Nevertheless, the lateral size of the resultant NSs remains highly restricted with a low aspect ratio. Therefore, achieving the synthesis of thin *c*-MOF NSs with high aspect ratios and superior flexibility remains a significant challenge.

Herein, we report a general MOF NS sacrifice approach (MNSA) to facilely synthesize benzene- and triphenylene-based *c*-MOF NSs, employing insulating MOF NSs as sacrificial precursors. The conversion from MOFs to *c*-MOF NSs was observed to follow a “localized conversion mechanism,” ensuring that the *c*-MOF NS samples retained the morphology of the sacrificial precursors. Of particular note are the thin *c*-MOF-Cu-BHT NSs (BHT = benzenehexathiol), characterized by a high aspect ratio (>100) and significantly lower Young’s modulus and hardness (0.98 ± 0.20 and 0.09 ± 0.01 GPa, respectively) compared to bulk-type NPs (10.79 ± 1.30 and 0.75 ± 0.11 GPa, respectively). These properties facilitate the robust adhesion of Cu-BHT NSs to the gold electrode, thereby bolstering the long-term stability of the device, wherein the corresponding chemiresistive sensor displayed a coefficient of variation of only 0.5% over ten consecutive cycles. The Cu-BHT NS chemiresistive nanosensor demonstrates a substantial 47.1-fold improvement in the signal-to-noise ratio when exposed to acetone at room temperature compared to its bulk-type counterpart. Integrated with highly efficient machine learning techniques, the high-performance Cu-BHT NS-based chemiresistive sensor exhibits excellent gas identification performance (accuracy 97.9%) to multiple gas components (AC, FDH, EG, and PX), superior to those of previously reported chemiresistive sensors. Our work provides unique opportunities to design high-aspect-ratio and flexible *c*-MOF NSs as electrode materials for electronic devices.

## Results and Discussion

### Synthesis and Characterization

**Figure 1:**
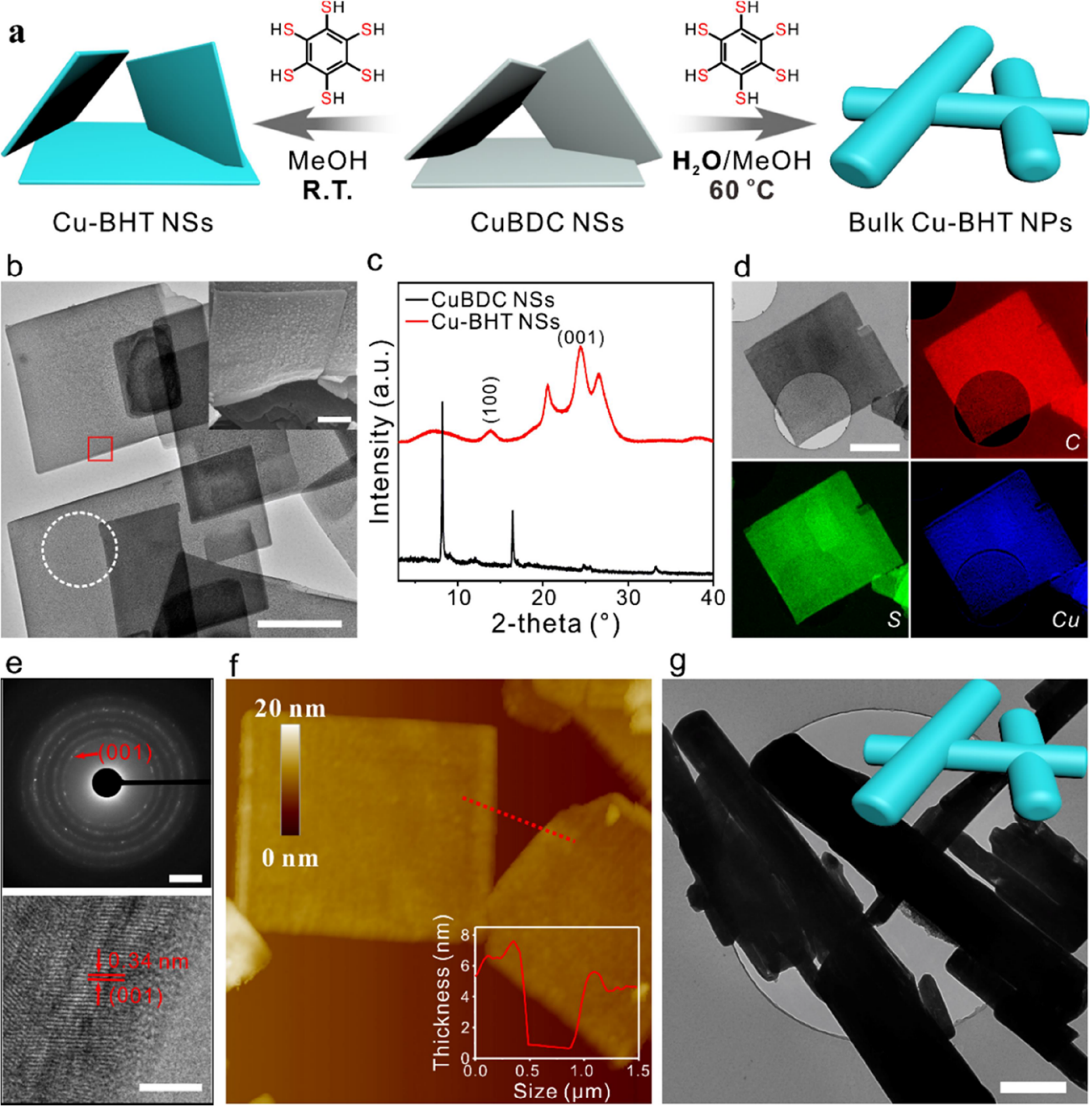
(a) Schematic overview of the synthesis of Cu-BHT NSs and bulk-type Cu-BHT NPs. (b) SEM image of Cu-BHT NSs. Inset: SEM image of Cu-BHT NSs. (c) TEM image of Cu-BHT NSs. (d) Powder XRD patterns. (e) SAED pattern (white circle in (e)) and HRTEM image of the red square shown in (e). (f) EELS mapping of Cu-BHT NSs. (g) Tapping-mode AFM image of Cu-BHT NSs and the height profile (inset) measured along the corresponding tracks shown in the atomi*c-*force micrograph. Scale bars represent 1 μm for (b), 200 nm for the inset in (b), 1 μm for (d), 2 1/nm for the top in (e), 5 nm for the bottom in (e), and 500 nm for (g).

The Cu-BHT NSs were synthesized based on an MNSA using copper 1,4-benzenedicarboxylate (CuBDC) MOF as a sacrificial precursor (Figure 1a). The freestanding CuBDC NSs were first synthesized as described previously^26^ and feature thin square lamellae structures with the average size of 0.5–4.0 μm (Supplementary Figure S1). After a 1 h reaction with BHT ligands, the light-blue CuBDC NSs completely evolved into dark Cu-BHT NSs in the solution (Figure 1b,c, Supplementary Figures S2 and S3). High-magnification SEM and TEM images reveal the resulting Cu-BHT NSs maintained the morphology of the sacrificial precursor, yet their surface becomes uneven with numerous defects (Supplementary Figure S2). Elemental mapping by the corresponding low-loss electron energy loss spectroscopy (EELS) indicated that the C, S, and Cu elements were homogeneously distributed over the whole Cu-BHT NSs (Figure 1d). The polycrystalline feature of Cu-BHT NSs was verified by the selected-area electron diffraction (SAED) pattern (Figure 1e). High-resolution TEM (HRTEM) imaging further indicated the polycrystalline Cu-BHT NSs with a lattice spacing of 0.34 nm, which was attributed to the *d*-spacing of the (001) plane of Cu-BHT (Figure 1e).^27^ The atomic force microscopy (AFM) image depicts a square sheet structure with a thickness of ∼5 nm, resulting in aspect ratios exceeding 100 (Figure 1f). Interestingly, varying the solution and temperature (water fraction of 25% and 40 °C) allows the production of bulk-type Cu-BHT NPs (Figure 1g, Supplementary Figure S4). Unlike the insulating CuBDC precursor, Cu-BHT NSs display an intrinsic electrical conductivity of 0.79 S cm^–1^, while bulk Cu-BHT NPs boast an intrinsic electrical conductivity of 91.32 S cm^–1^ (Table S1).

The Cu-BHT NSs share identical crystalline structures with bulk Cu-BHT NPs (Supplementary Figure S5a). FT-IR spectroscopy, X-ray photoelectron spectroscopy, and thermogravimetric analyses further confirmed the identical compositions of these Cu-BHT samples (Supplementary Figure S5b–d). The Brunauer–Emmett–Teller (BET) measurements (Supplementary Figure S6) revealed that the surface area of the Cu-BHT NS samples (98.1 m^2^g^–1^) was over 6.0 times higher than that of the Cu-BHT bulk samples (16.1 m^2^g^–1^).

### Transformation Mechanism

During the transformation, the driving force is supposed to be the formation of more stable coordination bonds. The CuBDC structure exhibited poor water stability because weak metal–oxygen coordination can be irreversibly degraded by water^28^ (Supplementary Figures S7a and S8a). However, the Cu-BHT with square planar CuS_4_ building units exhibits excellent chemical stability, and the Cu-BHT NSs can retain high crystallinity under harsh solutions (concentrated 10 M H_2_SO_4_, Supplementary Figures S7b and S8b). During the transformation, the CuBDC template with weaker Cu–O coordination bonds was decomposed and the Cu-BHT with stable coordination Cu–S bonds was established.

**Figure 2:**
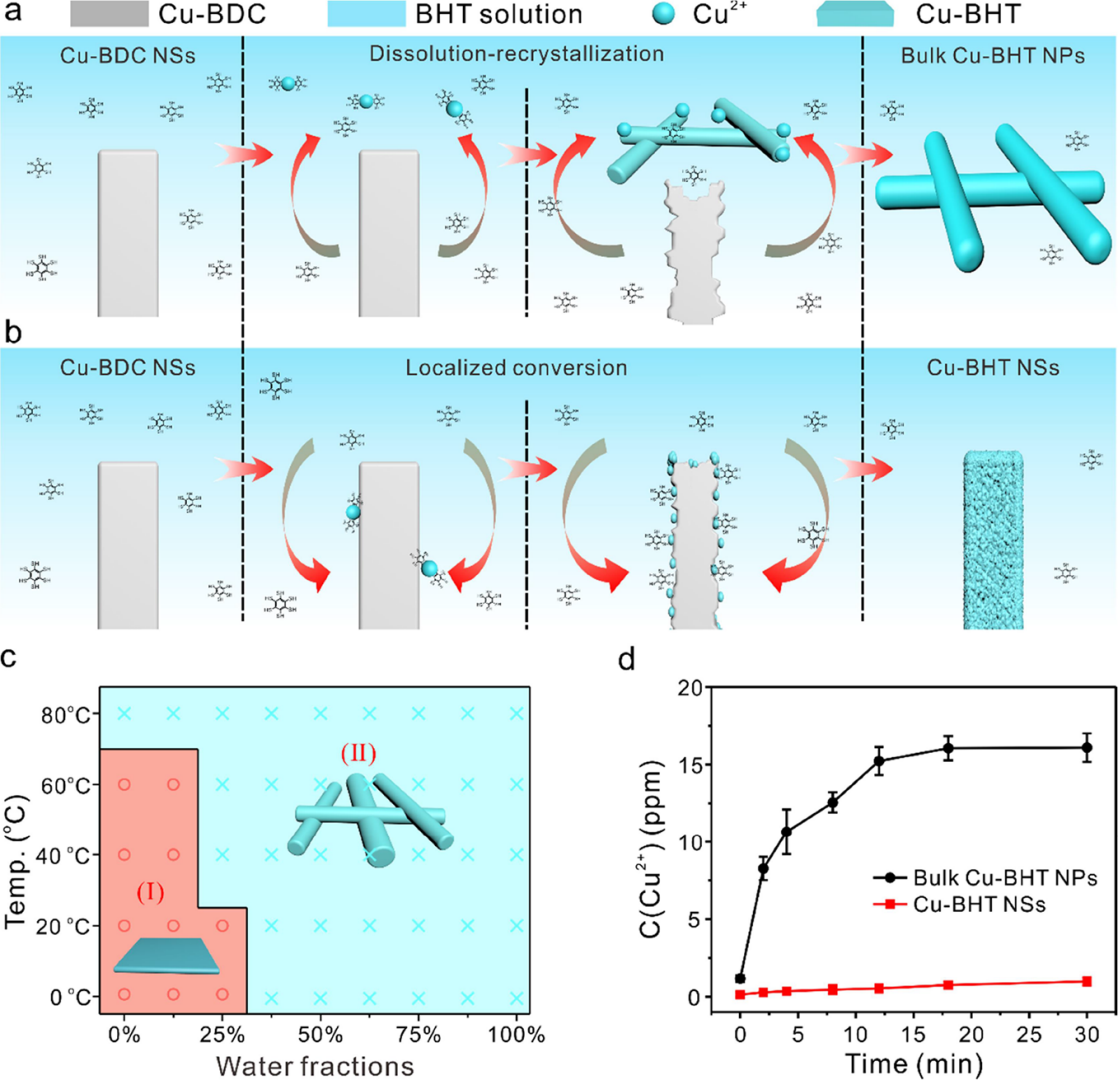
Transformation mechanism. (a) Schematic overview of the transformation of CuBDC NSs to bulk Cu-BHT NPs. (b) Schematic overview of the transformation of CuBDC NSs to Cu-BHT NSs. (c) Phase diagram that correlates the solvent composition (horizontal ordinate) and reaction temperature (vertical ordinate). Zone (I) is Cu-BHT NSs; zone (II) is bulk Cu-BHT NPs. (d) Concentration of Cu^2+^ in the reaction solution versus reaction time toward the construction of Cu-BHT NSs and Cu-BHT bulk, respectively.

During the MNSA transformation, our observation reveals that the morphologies of the final products significantly depend on the reaction kinetics. By tailoring the reaction kinetics of the CuBD*C-*to-Cu-BHT conversion via water content and temperature (Figure 2a–c), we could achieve NS-like and rod-like Cu-BHT samples. The Cu-BHT NSs can only be obtained at low temperatures and water fractions (e.g., 20 °C and 12.5%), while higher reaction temperatures and water fractions (e.g., 60 °C and 75%) resulted in the formation of bulk-type samples. Based on the above experimental results, a possible reaction path is proposed to illustrate the transformation from CuBDC to Cu-BHT. The total reaction can be represented as follows:
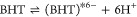
1
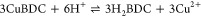
2
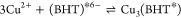
3

The total reaction is represented as follows:
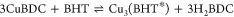
4

The formation of bulk Cu-BHT NPs in a methanol/water solution (v:v = 3:5) is proposed to follow a “dissolution-recrystallization mechanism”^29−31^ (Figure 2a). Due to the rapid dissolution of CuBDC at high water fractions, a significant number of Cu^2+^ cations were quickly released into the reaction solution and coordinated with BHT ligands (eqs 1 and 2, Figure 2d). Over time, Cu-BHT nucleated from the supersaturated solution and deposited onto the surface of the BHT ligand templates (eq 3). In contrast, the formation of Cu-BHT NSs in methanol solution is suggested to proceed via a “localized conversion mechanism”^32,33^ (Figure 2b). In this process, the reaction sites are strongly localized within the CuBDC template, accompanied by recrystallization. At low temperatures and water fractions, the concentration of Cu^2+^ cations in the solution remains low, preventing Cu_3_(BHT*) nuclei from reaching the critical nucleation concentration required for Cu-BHT crystal formation (Figure 2d). As a result, eq 3 does not occur under these conditions, and the conversion process predominantly takes place on the surface of CuBDC NSs through a direct heterogeneous reaction (eq 4).

**Figure 3:**
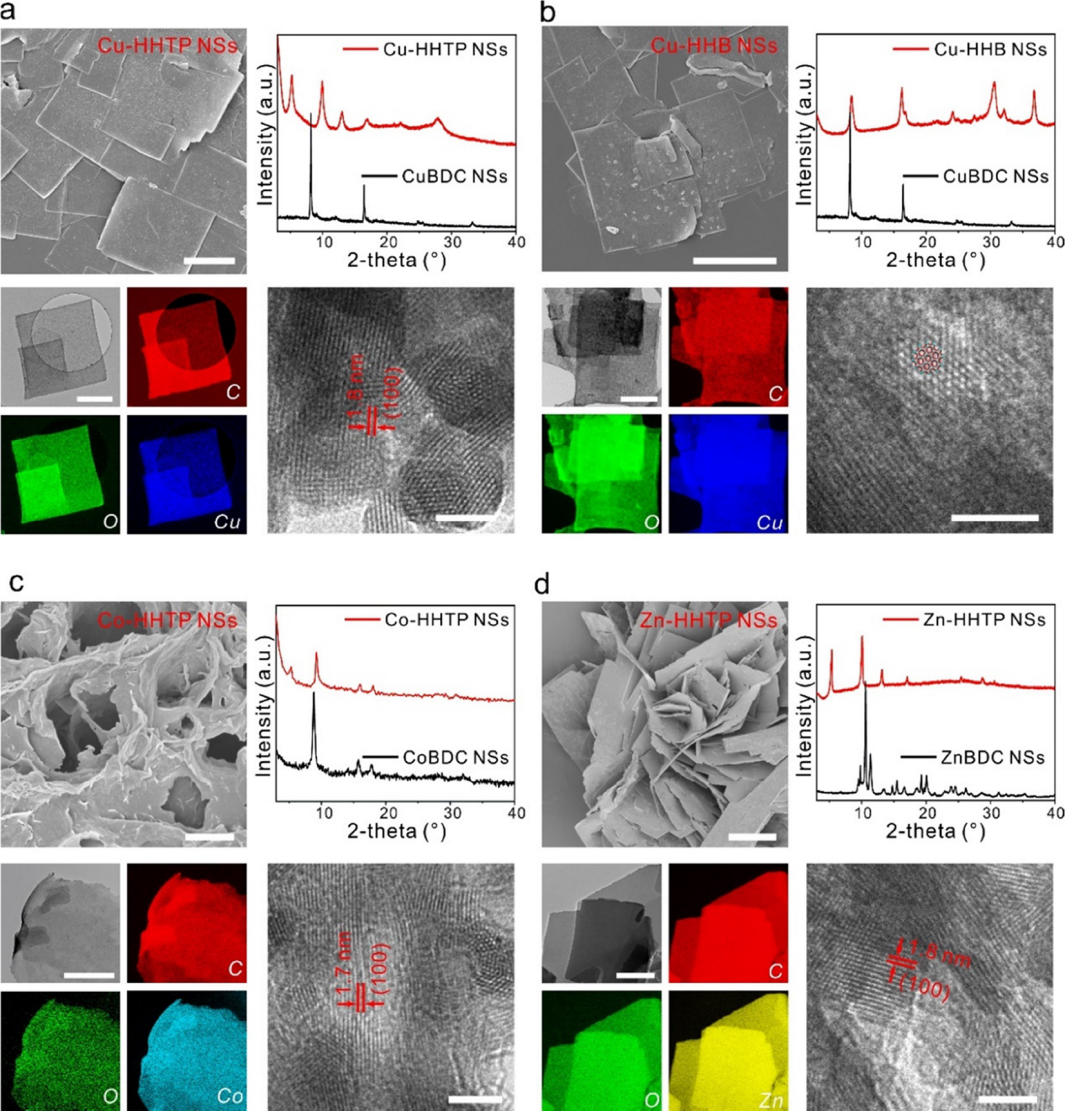
Versatility of the synthesis strategy to produce *c*-MOF NSs. (a) Characterizations of Cu-HHTP NSs. (b) Cu-HHB NSs. (c) Characterizations of Co-HHTP NSs. (d) Characterizations of Zn-HHTP NSs. Top left: SEM image, and scale bars represent 1 μm for (a–d); bottom left: elemental mapping image, and scale bars represent 500 nm for (a–d); top right: X-ray diffraction pattern; bottom right: HRTEM image, and scale bars represent 20 nm for (a–d). To broaden the applicability of the MNSA strategy, we systematically prepared a range of *c*-MOF NSs by substituting Cu^2+^ with alternative metal nodes (Co^2+^ and Zn^2+^) or by replacing BHT with alternative conjugated linkers (2,3,6,7,10,11-hexahydroxytriphenylene (HHTP) and hexahydroxybenzene (HHB)) (Figure 3, Supplementary Figure S9, Tables S1 and S2). Upon 1 h treatment at room temperature, all of the MOF NS sacrificial precursors (CuBDC, CoBDC, and ZnBDC) were completely converted into highly crystalline *c*-MOF NSs (Cu-HHTP, Cu-HHB, Co-HHTP, and Zn-HHTP), as verified by XRD patterns. The homogeneous spatial distributions of the metal elements and oxygen in the NSs were also confirmed by EDS elemental mappings. Both scanning electron microscopy and atomic*-*force microscopy revealed sheet structures with lateral dimensions ranging from 0.5 to 2 μm and thicknesses spanning from 7 to 30 nm, resulting in aspect ratios ranging from 15 to 70. Notably, no surfactants were used during the synthesis process. Collectively, these results highlight the versatility of the MNSA synthesis methodology in producing *c*-MOF NSs.

### Mechanical Properties and Device Performance

**Figure 4:**
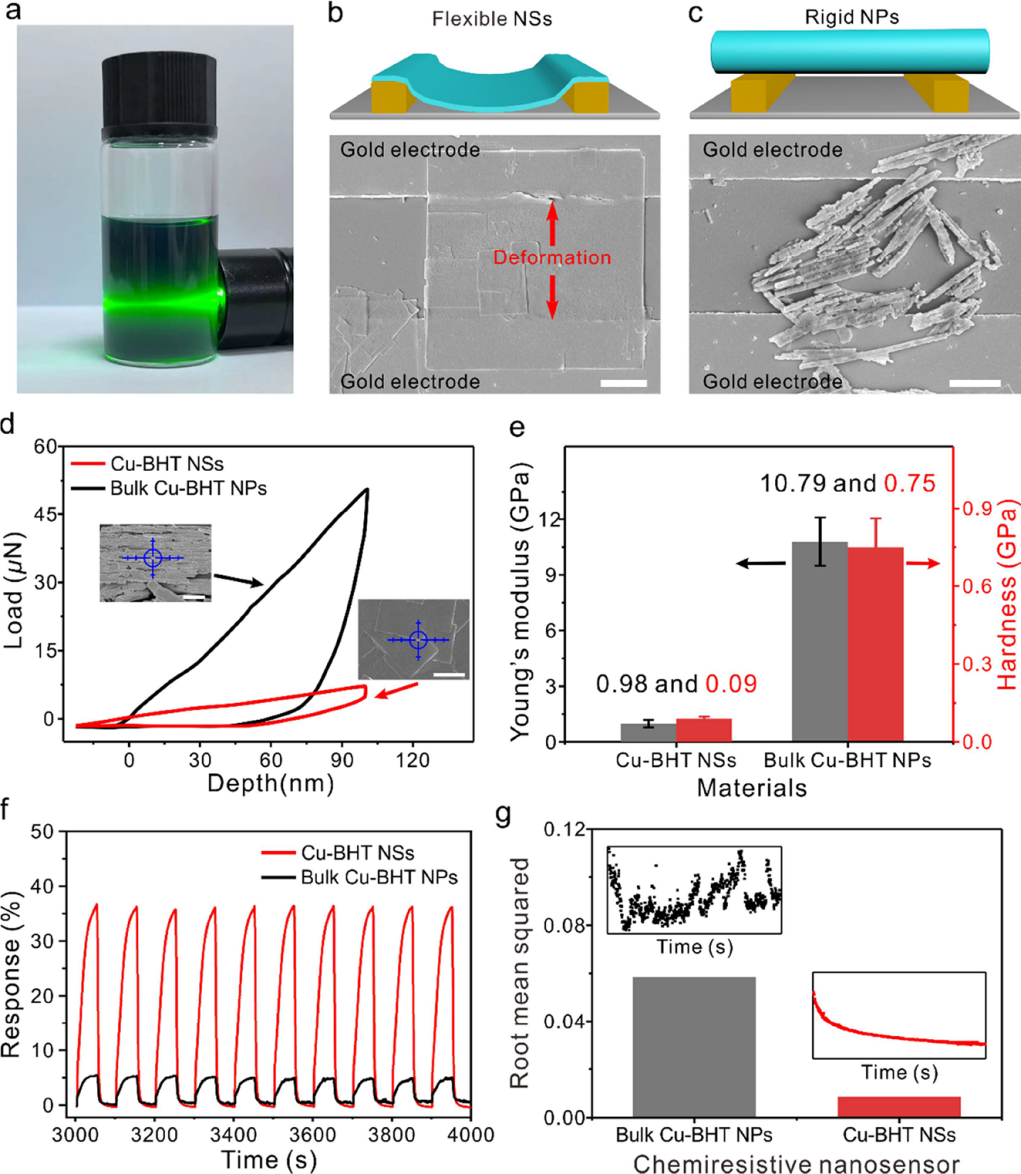
Sensing performance and signal processing. (a) Schematic overview of the Cu-BHT-based electronic devices. (b) Schematic overview of the flexible Cu-BHT NSs deposited on IDEs (top) and the SEM image of Cu-BHT NSs deposited on IDEs for gas sensing. (c) Schematic overview of the rigid bulk Cu-BHT NPs deposited on IDEs (top) and SEM image of bulk Cu-BHT NPs deposited on IDEs for gas sensing. (d) Typical load–displacement nanoindentation curves for different MOF film structures. The insets are SEM images of the surfaces of the bulk-type Cu-BHT and the NS film on silicon wafers, respectively; the blue crosshair represents the test site. (e) Young’s modulus and hardness of different Cu-BHT structures. (f) Response of Cu-BHT-based sensors toward 200 ppm acetone at room temperature. (g) Comparison of root-mean-squared (RMS) value—representing the noise-based deviation in response—was calculated using the baseline trace before exposure to acetone. Scale bars represent 1 μm for (b), 2 μm for (c), and 1 μm for (d).

The well-dispersed Cu-BHT NSs with significant Tyndall effect can be facilely transferred and deposited on interdigital electrode (IDE) devices to construct a Cu-BHT NS-based chemiresistive sensor by using the Langmuir–Schäfer method^34,35^ (Figure 4a, Supplementary Figures S11 and S12). As shown in Figure 4b, the flexible Cu-BHT NSs can adapt their shape to conform to the channels of the electrodes, significantly enhancing the effective contact surface between the NSs and the electrode and thus ensuring strong adhesion to the electrode substrate. In contrast, the bulk Cu-BHT NPs demonstrate weak adhesion interactions with the electrode substrate due to their rigid structure induced limited effective contact surface, suggesting potential detachment from the IDE device (Figure 4b). This should be attributed to the distinct mechanical strength between Cu-BHT NSs and bulk-type Cu-BHT NP as the NSs have significantly lower strength compared to bulk NPs because their surface atomic coordination and cohesion are weaker.^36^

The mechanical properties of Cu-BHT samples were measured by an AFM nanoindenter equipped with a tip radius of 100 nm. In a typical load–displacement cycle, the peak load was enhanced up to ≈50.54 μN for the bulk-type film, which was over 7.0 times larger than that of the Cu-BHT NS film (≈7.17 μN) at the same indentation depth of 100 nm (Figure 4d). The Young’s modulus and hardness of Cu-BHT NS were obtained to be 0.98 ± 0.20 and 0.09 ± 0.01 GPa, respectively, which were only ∼10% of those of the bulk-type Cu-BHT NPs (10.79 ± 1.30 and 0.75 ± 0.11 GPa, Figure 4e). This flexibility enables the Cu-BHT NSs to alter their morphology and readily adhere to electrodes, providing significant advantages for their stability in device applications.

The chemiresistive gas-sensing properties of Cu-BHT NS- versus bulk Cu-BHT NP-based chemiresistors toward acetone at room temperature are demonstrated in Figure 4f. The response intensity of Cu-BHT NSs to 200 ppm acetone was 36.28 ± 0.18%, which represents a 6.95-fold improvement compared to the bulk-type Cu-BHT NPs (5.22 ± 0.21%). This excellent gas-sensing performance of the Cu-BHT NSs can be attributed to their increased BET surface area (16.1 m^2^g^–1^ vs 98.1 m^2^g^–1^) for more efficient gas enrichment, their ultrathin 2D NS morphology for faster mass transport, and a higher utilization ratio of inner active sites. Additionally, the Cu-BHT NS-based device exhibits exceptional repeatability with a coefficient of variation of merely 0.5% across ten consecutive cycles, outperforming the bulk Cu-BHT NP-based device, which registers a coefficient of variation of 4.0%. Prior to exposure to the analyte, the RMS deviation value—representing the noise-based deviation in response intensity—for the bulk-type Cu-BHT NP-based chemiresistor is approximately 7.0 times higher than that of the Cu-BHT NS-based nanosensor (Figure 4g). Therefore, the Cu-BHT NS-based nanosensor exhibits a 47.1 times higher signal-to-noise ratio (calculated by dividing the response intensity by the quadrature sum of the RMS noise) than that of the bulk-type Cu-BHT NP-based chemiresistor.

### Multiple Gas Component Screening

**Figure 5:**
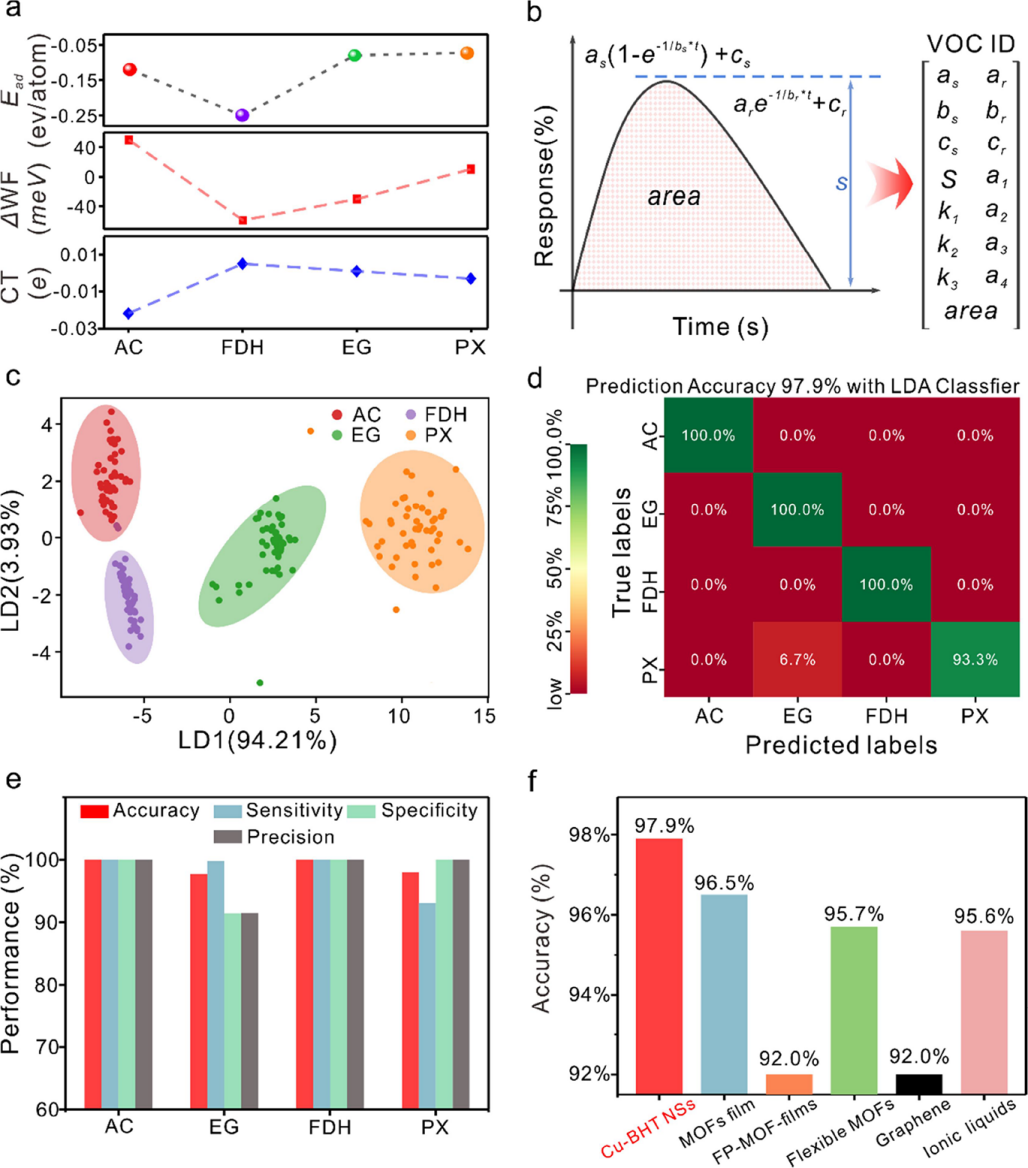
Gas identification mechanism and performance. (a) Adsorption energy (*E*_ad_, ev/atom), work function (ΔWF, meV), and charge transfer (CT, e^–^) of analyte gases upon interaction with sensing element materials Cu-BHT NSs implemented by DFT calculations. (b) Scheme of feature extraction strategy to each analyte gas; eventually, each analyte gas is represented by 15 transient parameters from the normalized sensing response profile. (c) LDA score plot for FDH, AC, EG, and PX analyte gases in the 2D space. (d) Confusion matrix result for prediction of four analyte gases using the hold-out validation approach. (e) VOC gas classification performance results of the developed chemiresistive sensors by the LDA Classifier algorithm using the hold-out validation approach. (f) Comparison with other reported electronic noses toward VOC discrimination at room temperature.

The high-performance Cu-BHT NS-based chemiresistive nanosensor can be applied to detect and discriminate multiple gas components, including acetone (AC), formaldehyde (FDH), ethylene glycol (EG), and p-xylene (PX) (Table S3). The sensing signal of Cu-BHT NS-based chemiresistive sensor upon exposure to the four VOCs is presented in Supplementary Figures S13 and S14. With the same experimental protocols, the average response amplitude of the same chemiresistive sensor varies from analyte gases and generally follows the order FDH > AC > EG > PX (Supplementary Figure S14). DFT calculations were introduced to investigate the adsorption energy (*E*_ad_), work function (Δ_WF_), and charge transfer (CT) for understanding the sensing-performance mechanisms (Supplementary Figure S15). Upon interaction with Cu-BHT NSs, FDH molecules exhibit the lowest adsorption energy, while EG and PX molecules show the highest (Figure 5a). Lower adsorption energy indicates a higher likelihood of gas molecules trapping and attaching to Cu-BHT, correlating with sensing response amplitudes. Despite AC analyte molecules showing the highest charge transfer, their sensing response is not significant compared to FDH analyte molecules, likely due to fewer binding molecules. Analyte gas molecules’ adsorption energy, charge transfer, and morphology differ distinctly, yielding unique VOC fingerprint characteristics for efficient machine learning algorithms (Figure 5b).

To demonstrate the identification performance for analyte gases using the above 15 transient features as shown in Figure 5b, a typical supervised machine learning classifier, linear discriminant analysis (LDA) algorithm, was utilized to classify analyte gases. As shown in the 2D LDA score plot and the 3D LDA score plot (Figure 5c, Supplementary Figure S16), it is observed that these four analyte gases form four individual clusters separately from each other with rather less overlapping. The first three linear discriminants account for 100% of the total variance (LD1, LD2, and LD3 explain 89.83, 9.62, and 0.55%, respectively), which indicates that these four analyte gases could be well classified. The classification confusion matrix is derived and is illustrated in Figure 5d. Most of the analyte gases could be classified correctly, while minor data of PX analyte vapors are misclassified into EG analyte vapors, resulting in an excellent overall accuracy of 97.9%, an overall sensitivity of 98.3%, and an overall specificity of 99.3% as shown in Figure 5e, which surpass the reported electronic nose based on MOFs, graphene, and ionic liquid composites in a much simpler and more portable structure (Figure 5f).^37−41^Supplementary Figure S17 presents the identification accuracy of these four analyte gases with respect to various classifier algorithms using the k-fold cross-validation approach (*k* = 10). For most of the classifier algorithms, the achieved prediction accuracy is higher than 94.0%, implying the high efficiency of VOC feature parameters. These results indicate the synergy effects of both the Cu-BHT NS material and these 15 effective feature parameters for the identification of these four analyte gases.

## Conclusions

In summary, we have implemented an efficient general MNSA strategy to synthesize thin *c*-MOF NSs, which involves a “localized conversion mechanism.” Constructing *c*-MOFs into thin NS nanostructures enhances both aspect ratios and the flexibility for intimate device integration. Integrated into chemiresistive devices, Cu-BHT NS-based sensors show superior device stability and signal-to-noise ratio for gaseous analytes at room temperature compared to bulk-type Cu-BHT NP-based sensors. Leveraging the distinctive fingerprint features of different analyte gases, the Cu-BHT NS-based chemiresistive sensors exhibit excellent gas identification performance for multiple gas components, surpassing previously reported electronic noses. We anticipate this work will not only stimulate the development of *c*-MOFs NS synthesis but also advance the functionalities of *c*-MOFs for electronic device applications.

## Materials and Methods

### Synthesis of CuBDC NS Precursors

The 3D MOF NSs were synthesized via a three-layer synthesis strategy as described previously.^26^ Typically, CuBDC NSs were synthesized in a glass vial. 30 mg of H_2_BDC was dissolved in a mixture of 2 mL of DMF and 1 mL of CH_3_CN and was poured into the bottom of the vial. Over this solution, a mixture of 1 mL of DMF and 1 mL of CH_3_CN was carefully added to prevent premature mixing of the two solutions containing the precursors. Finally, 30 mg of Cu(NO_3_)_2_·3H_2_O was dissolved in a mixture of 1 mL of DMF and 2 mL of CH_3_CN and was also carefully added to the vial as the top layer. The synthesis proceeded at 40 °C for 24 h without any disturbance, and the resulting precipitate was collected by centrifugation and consecutively washed three times with methanol. The resulting material was left suspended in methanol to make a stock solution (∼5 mg/mL) for further use.

Similarly, zinc 1,4-benzenedicarboxylate (ZnBDC) was synthesized by using Zn^2+^ as a metal cation and H_2_BDC as an organic ligand. A linker solution composed of 20 mg of H_2_BDC dissolved in a mixture of 2 mL of DMF and 1 mL of CH_3_CN was employed as the bottom liquid layer, a mixture of 1 mL of DMF and 1 mL of CH_3_CN was the spacer layer, while a solution of 10 mg of Zn(CH_3_COO)_2_·2H2O in 1 mL of DMF and 2 mL of CH_3_CN was the top, metal-containing layer. Synthesis took place at 40 °C for 24 h under static conditions. Finally, the solid product was recovered by centrifugation and thoroughly washed as described previously for CuBDC NSs. The resulting material was left suspended in ethanol to make a stock solution (∼5 mg/mL) for further use.

Cobalt 1,4-benzenedicarboxylate (CoBDC) was synthesized by using Co^2+^ as a metal cation and H_2_BDC as an organic ligand. A linker solution composed of 10 mg of H_2_BDC dissolved in a mixture of 2 mL of DMF and 1 mL of CH_3_CN was employed as the bottom liquid layer, a mixture of 1 mL of DMF and 1 mL of CH_3_CN was the spacer layer, while a solution of 10 mg of Co(CH_3_COO)_2_·4H2O in 1 mL of DMF and 2 mL of CH_3_CN was the top, metal-containing layer. Synthesis took place at 25 °C for 24 h under static conditions. Finally, the solid product was recovered by centrifugation and thoroughly washed as described previously. The resulting material was left suspended in ethanol to make a stock solution (∼5 mg/mL) for further use.

### Synthesis of Cu-BHT NSs from CuBDC NS Precursors

Typically, 2 mL of as-synthesized CuBDC stock solution was dissolved in 3 mL of methanol to form a light-blue solution. A solution of 5 mg of BHT ligand in 5 mL of methanol was added to the CuBDC solution. The mixture was stirred at room temperature for 1 h. The dark precipitate was collected by centrifugation, washed with methanol three times, and dispersed in 2 mL of ethanol (∼2 mg/mL) for further use. For comparison, the bulk-type Cu-BHT NPs were synthesized similar to the preparation of Cu-BHT NSs; typically, 2 mL of as-synthesized CuBDC stock solution was dissolved in 3 mL of methanol to form a light-blue solution. A solution of 5 mg of BHT ligand in 5 mL of water was added to the CuBDC solution. The mixture was stirred at room temperature for 1 h. The dark precipitate was collected by centrifugation, washed with methanol three times, and dispersed in 2 mL of ethanol (∼2 mg/mL) for further use.

### Synthesis of Cu-HHTP NSs from CuBDC NS Precursors

Typically, 2 mL of as-synthesized CuBDC stock solution was dissolved in 3 mL of ethanol to form a light-blue solution. 5 mg of HHTP ligand in 5 mL of ethanol/water solution (4:1, v/v) was added to the CuBDC solution. The mixture was stirred at room temperature for 1 h. The dark precipitate was collected by centrifugation, washed with methanol three times, and dispersed in 2 mL of ethanol (∼2 mg/mL) for further use.

### Synthesis of Cu-HHB NSs from CuBDC NS Precursors

Typically, 2 mL of as-synthesized CuBDC stock solution was dissolved in 3 mL of ethanol to form a light-blue solution. 5 mg of HHB ligand in 5 mL of ethanol/water solution (4:1, v/v) was added to the CuBDC solution. The mixture was stirred at room temperature for 1 h. The dark precipitate was collected by centrifugation, washed with methanol three times, and dispersed in 2 mL of ethanol (∼2 mg/mL) for further use.

### Synthesis of Co-HHTP NSs from CuBDC NS Precursors

Typically, 2 mL of as-synthesized CoBDC stock solution was dissolved in 3 mL of ethanol to form a light-blue solution. 5 mg of HHTP ligand in 5 mL of ethanol/water solution (4:1, v/v) was added to the CoBDC solution. The mixture was stirred at room temperature for 1 h. The dark precipitate was collected by centrifugation, washed with methanol three times, and dispersed in 2 mL of ethanol (∼2 mg/mL) for further use.

### Synthesis of Zn-HHTP NSs from ZnBDC NS Precursors

Typically, 2 mL of as-synthesized ZnBDC stock solution was dissolved in 3 mL of ethanol to form a light-blue solution. 5 mg of HHTP ligand in 5 mL of ethanol/water solution (4:1, v/v) was added to the ZnBDC solution. The mixture was stirred at room temperature for 1 h. The dark precipitate was collected by centrifugation, washed with methanol three times, and dispersed in 2 mL of ethanol (∼2 mg/mL) for further use.

### Sensor Fabrication

The Cu-BHT NS sensor device was fabricated by the Langmuir–Schäfer method on gold IDEs fabricated on silicon wafers. The bulk-type Cu-BHT NP sensor device was fabricated by drop-casting a Cu-BHT NP droplet (10 μL dispersion) on gold IDEs fabricated on silicon wafers. Electrode fabrication was implemented utilizing a standard microfabrication process comprising photolithography, gold thermal evaporation, and lift-off, similar to our previously published work.^42^ IDE structure on the device features a gap size of 3 μm and a finger width of 4 μm.

### VOC Vapor Preparation

A bubbler evaporation platform was built to produce VOC vapor as well as deliver VOC vapor to the gas performance evaluation chamber. The flow rate of the carrier gas was tuned by a mass flow controller (MFC, type number: GF040, Brooks Instruments Company, USA). The source of both the carrier gas and the dilution gas was airflow, whose flow rate was controlled by an MFC.

### Sensing Measurement

The measurement system was a homemade gas-sensing setup and was applied to measure the electrical property of the sensor upon exposure to various vapors. Upon VOC vapor adsorbed by Cu-BHT NSs on the sensor, the electrical conductivity of the sensor shifted due to charge carrier transfer. A constant bias voltage (0.1 V) was applied to the sensor, and the sensor current was recorded by a source meter (Keithley 2602, Tektronix GmbH, Germany). Each test contains both the gas exposure phase and the gas flushing phase. In the analyte gas exposure phase (3 min), the analyte gas vapor was generated by the bubbling evaporation approach using airflow as the carrier gas (300 sccm) and dilution gas (1700 sccm), while in the analyte gas flushing phase (2 min), the flow rate of the flushing gas remains constant (2000 sccm).

### Signal Noise Characterization

The noise of the developed sensors was deduced from the root-mean-square deviation at the baseline following fifth-order polynomial fitting.

### Gas Classification by Supervised Machine Learning

Before feeding into clustering algorithms, feature data of analyte gases was processed using StandardScaler, MinMaxScaler, and L_2_ normalization algorithms. The transformed features were further applied to LDA for dimensionality reduction and classification. Each analyte gas is represented by 15 transient parameters from the normalized sensing response profile, such as exponential fitting parameters for gas exposure profile (*a*_*s*_*,b*_*s*_*,c*_*s*_), exponential fitting parameters for gas flushing profile (*a*_*r*_*,b*_*r*_*,c*_*r*_), maximum response amplitude (*S*), the area under curve (*area*), first derivative fitting parameters (*k*_1_,*k*_2_,*k*_3_) (in which *k*_1_ and *k*_3_ denote the first derivative value at *t* = 0 and *t* = 300 s, respectively, and *k*_2_ denotes the local minimum value at time range 50 s < *t* < 250 s), and second derivative fitting parameters (*a*_1_,*a*_2_,*a*_3_,*a*_4_) (in which *a*_1_ and *a*_4_ denote the second derivative value at *t* = 0 and *t* = 300 s, respectively, and *a*_2_ and *a*_3_ denote the local minimum value and local maximum value of the second derivative fitting curve at 50 s < *t* < 250 s).

### DFT Calculation

The interaction between gas molecules and MOFs was analyzed based on the framework of density functional theory (DFT) within the PBE generalized gradient approximation (GGA) for the exchange-correlation functional and the PAW method^43^ using the Vienna ab initio simulation package.^44,45^ The wave functions were expanded in plane waves up to a kinetic energy cutoff of 400 eV. The Brillouin zone was sampled by 2 × 4 × 1 k-points using the Monkhorst–Pack scheme.^46^ Periodic boundary conditions were applied for all calculations with the super cell size of about (15, 8.7, 30 Å). The dispersion corrections were included through the standard D2 Grimme parametrization.^47^ In order to figure out the most stable adsorption sites and molecular orientation, first-principles molecular dynamics simulations for 1 ps at room temperature were carried out using a canonical ensemble. Geometry optimizations were then performed for the most stable configurations. The adsorption energy is defined as the total energy difference of the substrate together with the adsorbate, the energy of the substrate, and the energy of an isolated molecule. In general, a higher adsorption energy implies a higher sensitivity for sensors.

## Supporting Information Available

Experimental methods, additional results, discussion, and characterization data (PDF)
