# Supplementary material for: Synthesizing Conductive Metal–Organic Framework Nanosheets for High-Performing Chemiresistive Sensors
Source: ACS Appl Mater Interfaces. 2025 Mar 15;17(12):18771–80. doi: 10.1021/acsami.5c00064 (PMC11956008; doi:10.1021/acsami.5c00064)
Supplement: Supplementary file 1 — am5c00064_si_001.pdf [file am5c00064_si_001.pdf]

Supporting Information

Synthesizing Conductive Metal-Organic Framework Nanosheets for High-Performing Chemiresistive Sensors

Chuanhui Huang[1+], Shirong Huang[2+*], Wei Wang[2+], Xing Huang[1], Arezoo Dianat[2], Rashid Iqbal[3], Geping Zhang[4,5], Naisa Chandrasekhar[1], Luis Antonio Panes-Ruiz[2], Yang Lu[1], Zhongquan Liao[6], Bergoi Ibarlucea[2], Chenchen Wang[1,2], Xinliang Feng[1,7], Gianaurelio Cuniberti[2,8*] and Renhao Dong[4,5*]

[1]Center for Advancing Electronics Dresden (Cfaed) and Faculty of Chemistry and Food Chemistry, Technische Universität Dresden, 01062 Dresden, Germany.

[2]Institute for Materials Science and Max Bergmann Center for Biomaterials, TUD Dresden University of Technology, 01062 Dresden, Germany. E-mail: shirong.huang@tu-dresden.de, gianaurelio.cuniberti@tu-dresden.de

[3]Key Laboratory of Colloid and Interface Chemistry of the Ministry of Education, School of Chemistry and Chemical Engineering, Shandong University, Jinan 250100, China.

[4]Department of Chemistry, The University of Hong Kong, Hong Kong 999077, China. E-mail: rhdong@hku.hk.

[5]Materials Innovation Institute for Life Sciences and Energy (MILES), HKU-SIRI, Shenzhen 518048, China

[6]Fraunhofer Institute for Ceramic Technologies and Systems (IKTS), Maria-Reiche-Strasse 2, 01109 Dresden, Germany.

[7]Department of Synthetic Materials and Functional Devices, Max Planck Institute for Microstructure Physics, D-06120 Halle (Saale), Germany.

[8]Dresden Center for Computational Materials Science (DCMS), TUD Dresden University of Technology, 01062 Dresden, Germany

[+] These authors contributed equally to this work

Method

Materials

All chemicals used were at least of analytical grade. Copper nitrate trihydrate (Cu $(NO_3)_2$•$3H_2O$), 1,4-benzenedicarboxylic acid (H_2BDC) were purchased from Sigma-Aldrich (Germany). Benzenehexathiol (BHT) purchased from BLD pharm company (Germany). Ultrapure water (18.2 MΩ) produced by a Millipore direct-Q system (Millipore) was used throughout the experiments. Commercial reagents were purchased from Sigma-Aldrich (ACS grade) and used as received unless otherwise noted.

Characterisation

Powder X-ray diffraction (PXRD) patterns were obtained on an X-ray diffractometer (Aeris Research Edition, Malvern Panalytical Company) using Cu-Kα radiation (λ = 0.15418 nm) at 40 kV and 15 mA at room temperature. The as-obtained powder samples were measured in reflection geometry. The FT-IR spectra were measured using a Bruker Tensor II IR spectrometer with a universal Zn-Se ATR (attenuated total reflection) accessory in the 500~4000 cm^{-1}. Scanning electron microscopy (SEM) was carried out on a field emission scanning electron microscope (FESEM, Zeiss Gemini 500). Transmission electron microscopy (TEM) measurements were carried out with a Libra120 (Carl Zeiss Microscopy GmbH, Germany). Transmission electron microscopy (TEM) and high-resolution TEM (HRTEM) were performed using a LIBRA 120 MC Cs STEM (Carl Zeiss) operating at an accelerating voltage of 120 kV. The surface area and pore diameter were determined with a physisorption analyser (model ASAP 2020M; Micromeritics, Norcross, GA, USA) at -196 °C. Before measurements, samples were degassed *in vacuo* at 100 °C for at least 8 h. The Brunner−Emmet−Teller (BET) method was used to calculate the specific surface areas (SBET) using adsorption data at P/P_0 of 0.05–0.30. The pore size distributions (PSDs) were derived from the adsorption branches of the isotherms using the Barrett-Joyner-Halenda (BJH) model. The total pore volume (V_t) was estimated from the adsorbed amount at P/P_0 of 0.995. Thermogravimetric analysis (TGA) was carried out using a Pyris 1 TGA (PerkinElmer, Waltham, MA, USA) with a nitrogen flow of 10 mL min^{-1}.

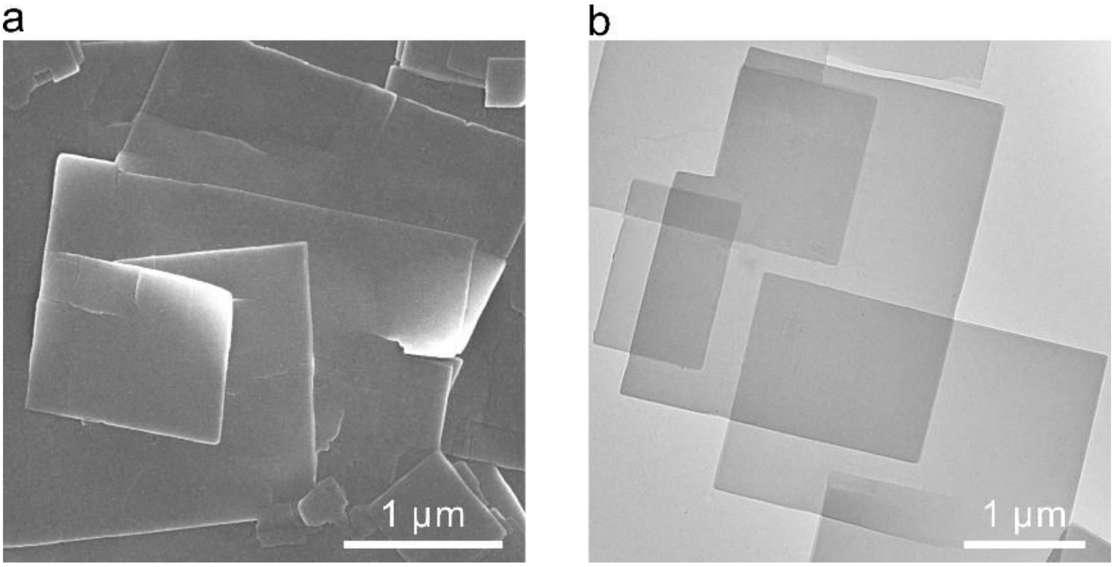

Figure S1. (a, b) SEM and TEM images of CuBDC NSs. Scale bars, 1 μm for (a, b).

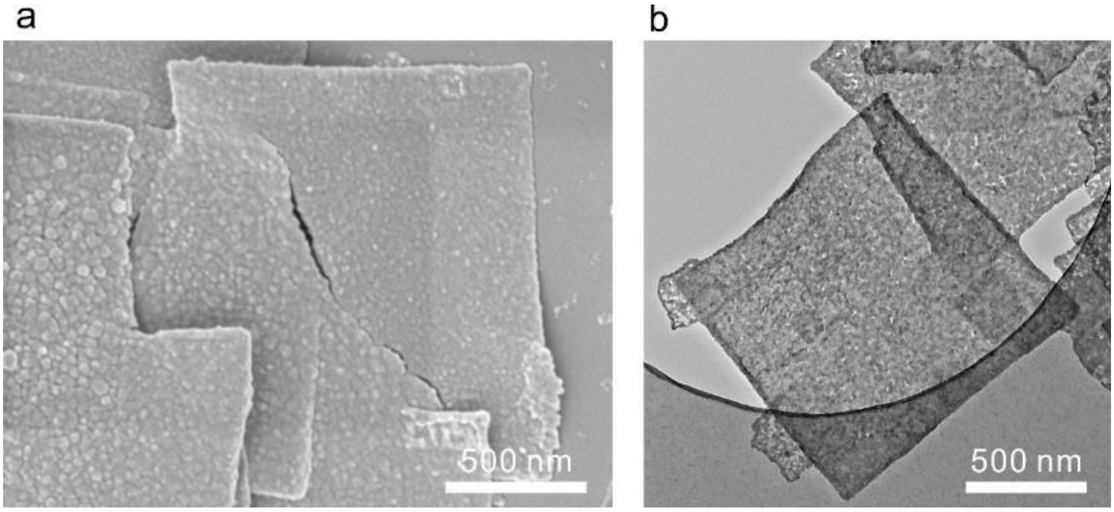

Figure S2. (a, b) SEM and TEM images of Cu-BHT NSs. Scale bars, 500 nm for (a, b).

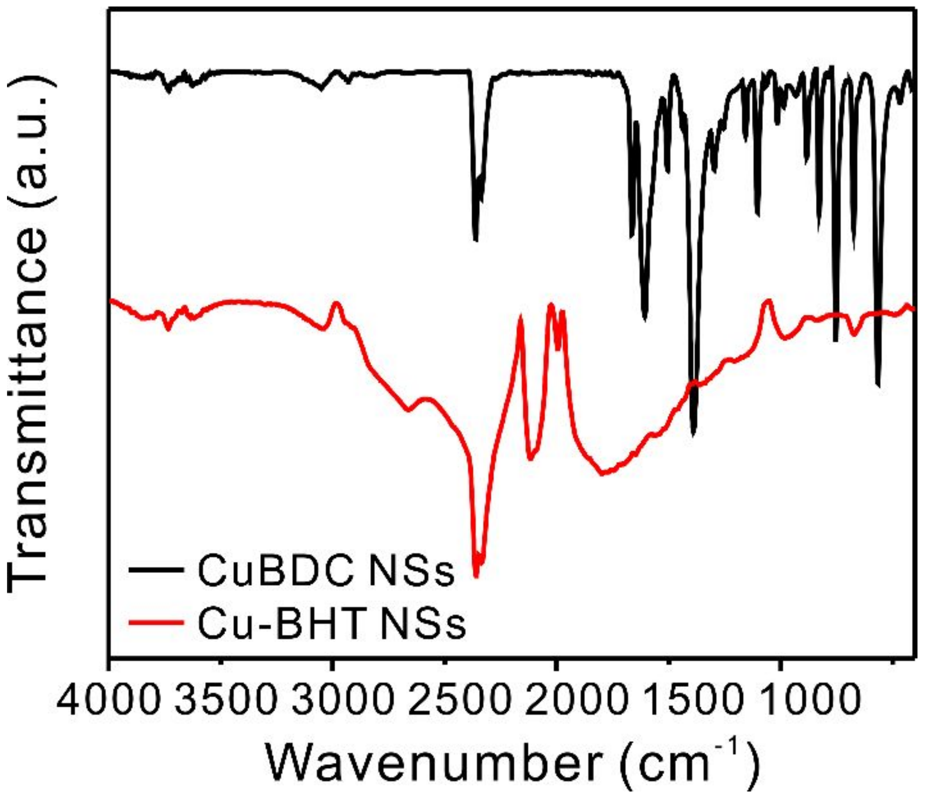

Figure S3. FT-IR spectra of the transformation from CuBDC NSs to Cu-BHT NSs.

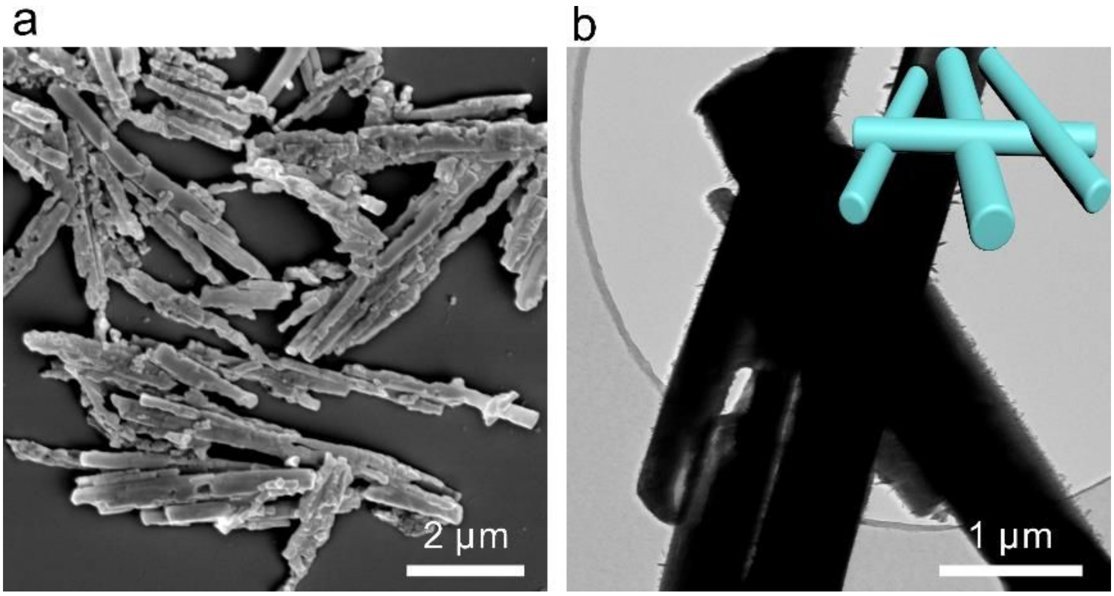

Figure S4. (a) SEM and (b) TEM images of bulk Cu-BHT NPs.

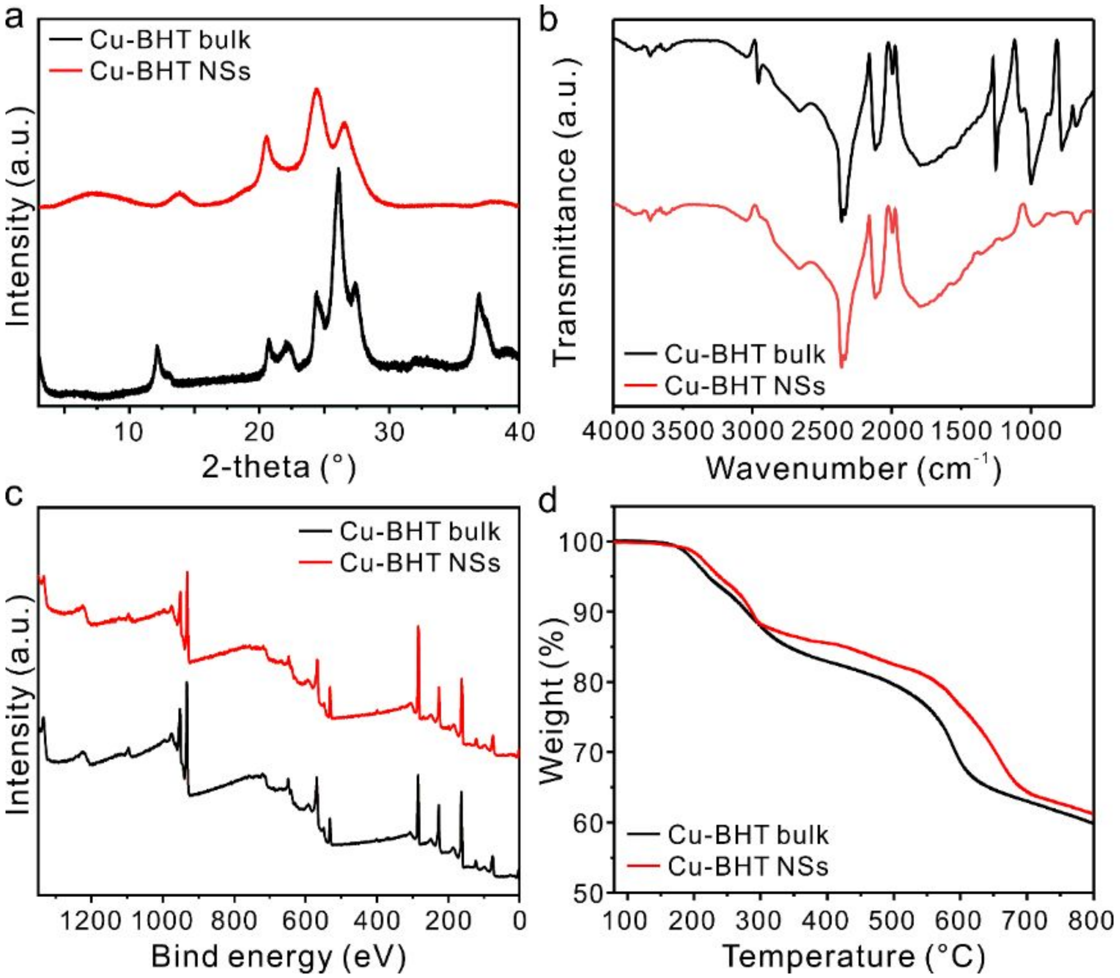

Figure S5. (a) Powder XRD patterns of the Cu-BHT samples. (b) FT-IR spectra of as-synthesized Cu-BHT samples. (c) XPS patterns of as-synthesized Cu-BHT samples. (d) TGA curves of as-synthesized Cu-BHT samples.

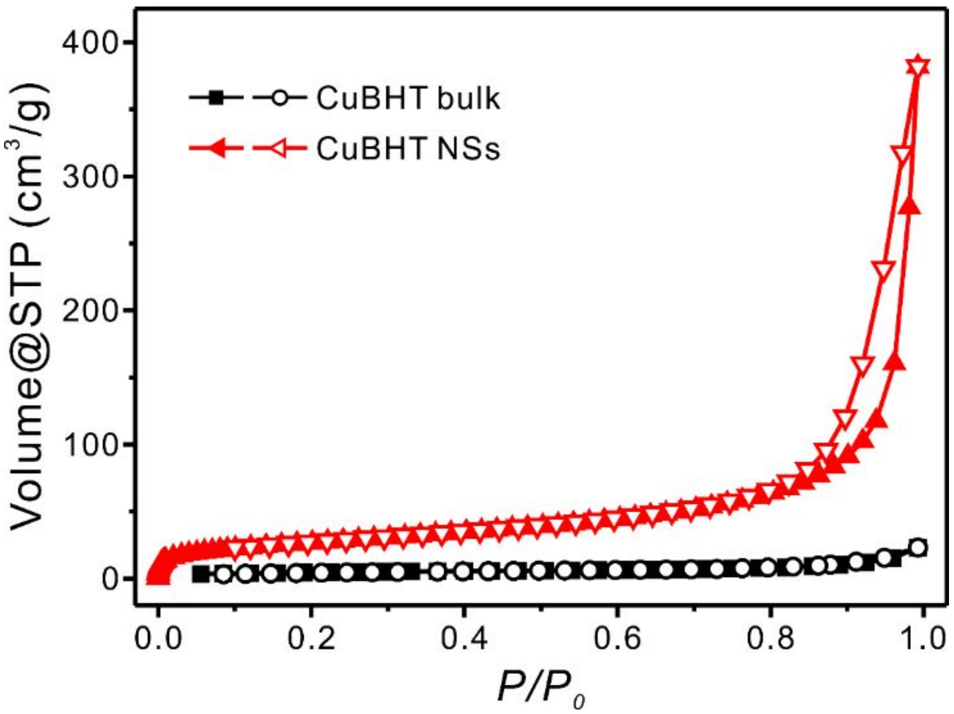

Figure S6. Nitrogen adsorption and desorption isotherms measured at 77 K of as-synthesized Cu-BHT samples.

The BET surface area of Cu-BHT bulk and Cu-BHT NSs are 16.1 and 98.1 $m^2\ g^{-1}$, respectively.

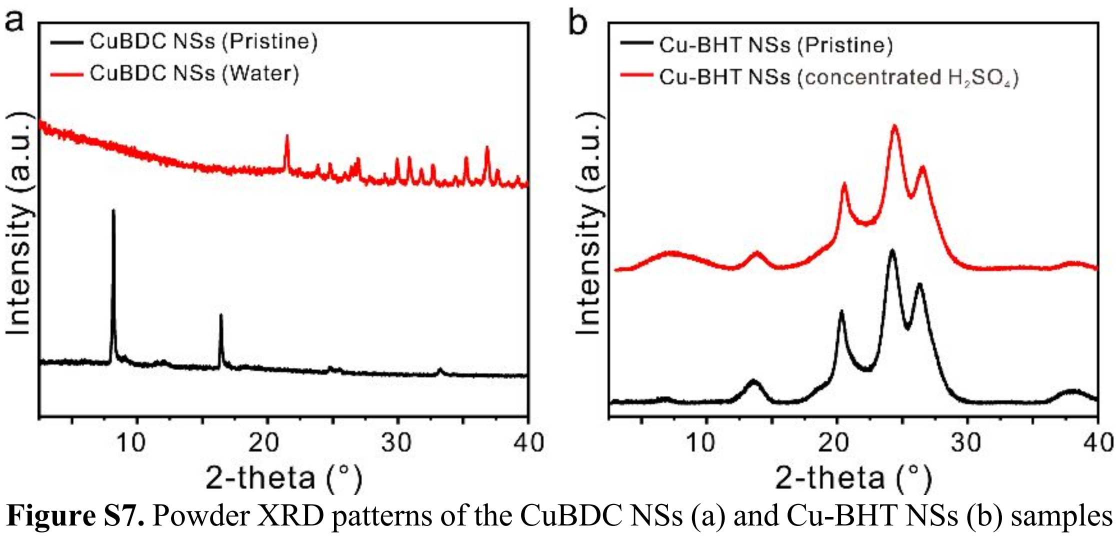

Figure S7. Powder XRD patterns of the CuBDC NSs (a) and Cu-BHT NSs (b) samples soaked at different solution conditions.

The chemical stability of Cu-BHT NSs was tested in concentrated H_2SO_4 (10 M). The PXRD indicated that Cu-BHT NSs retained high crystallinity under harsh solution (concentrated H_2SO_4 solution).

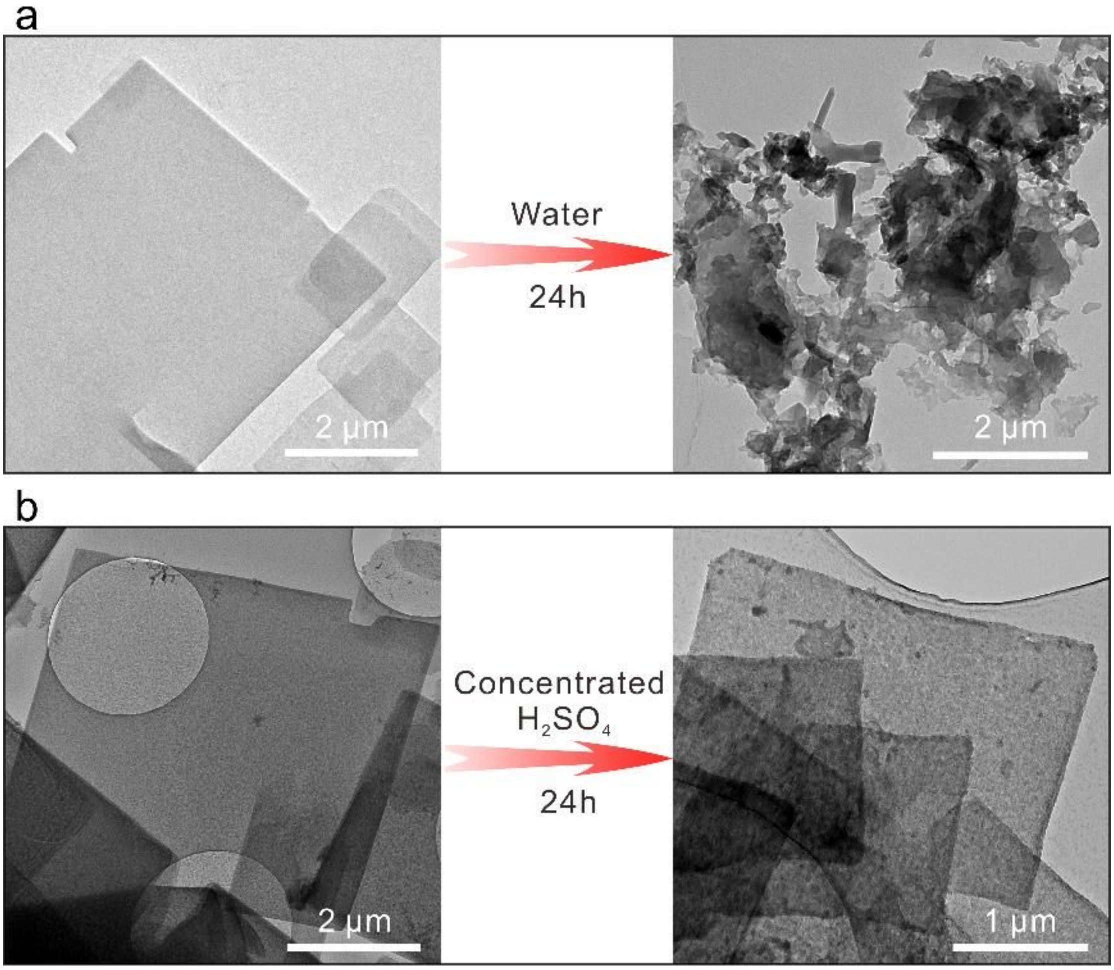

Figure S8. (a) TEM image of CuBDC NSs before and after soaking in water for 24 h. (b) TEM image of Cu-BHT NSs before and after soaking in concentrated H_2SO_4 (10 M) for 24 h.

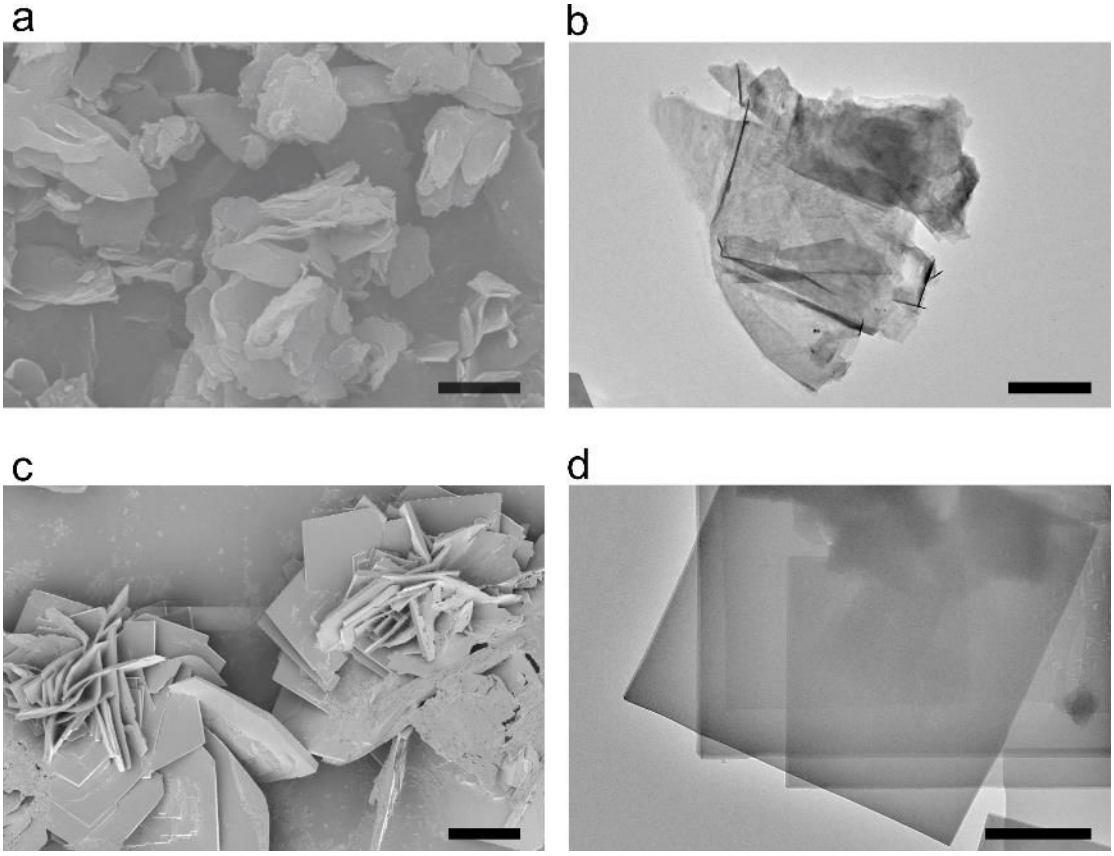

Figure S9. (a, b) SEM and TEM images of CoBDC NSs. (c, d) SEM and TEM images of ZnBDC NSs. Scale bars, 1 μm for (a-d).

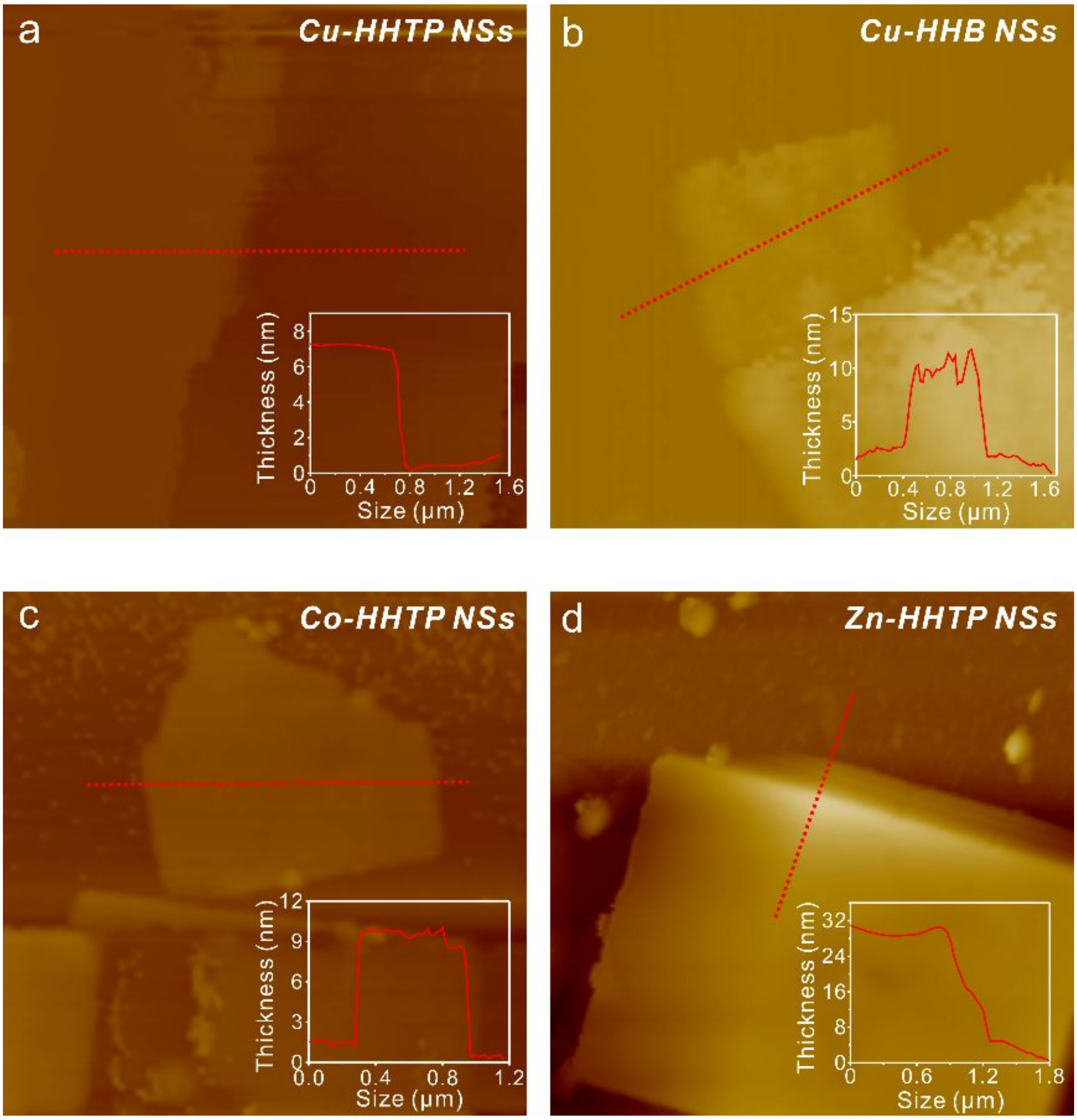

Figure S10. (a) AFM image of Cu-HHTP NSs and the height profile along the marked lines in the image. (b) AFM image of Cu-HHB NSs and the height profile along the marked lines in the image. (c) AFM image of Co-HHTP NSs and the height profile along the marked lines in the image. (d) AFM image of Zn-HHTP NSs and the height profile along the marked lines in the image.

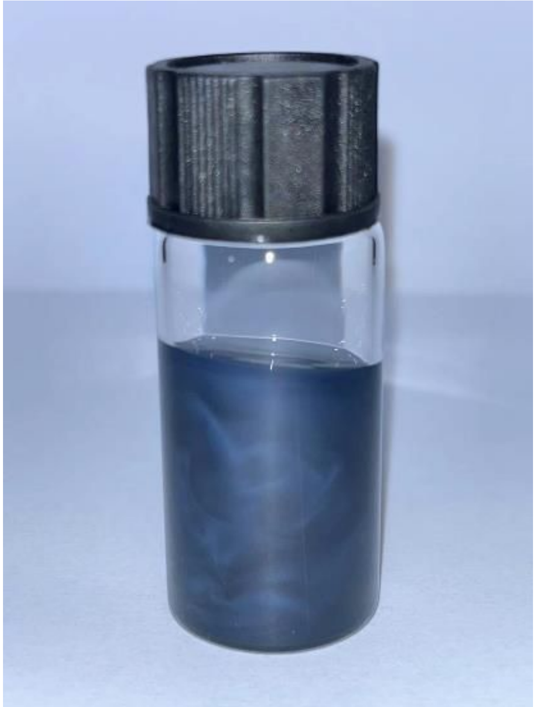

Figure S11. The optical photograph of Cu-BHT NSs in methanol solution.

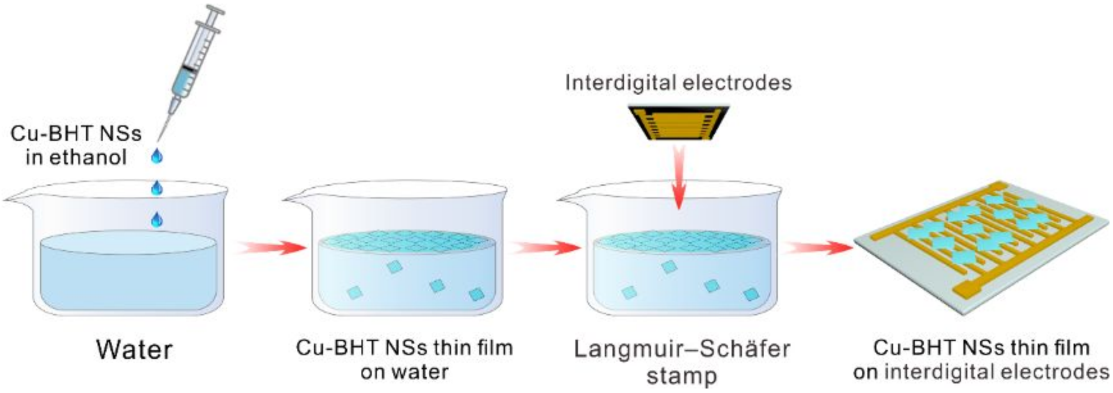

Figure S12. Schematic illustration of the assembly process for preparation of Cu-BHT NSs-based thin film on interdigital electrodes by Langmuir–Schäfer method.

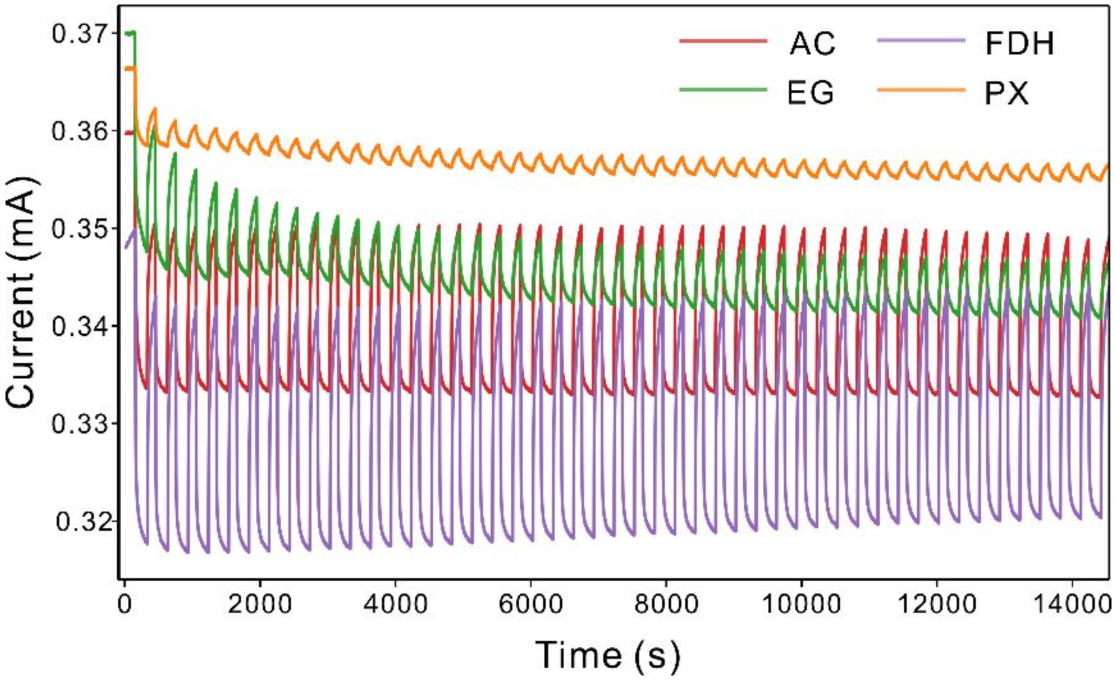

Figure S13. Raw sensing signal profile of Cu-BHT NSs-based nanosensors upon exposure to four types of analyte vapors.

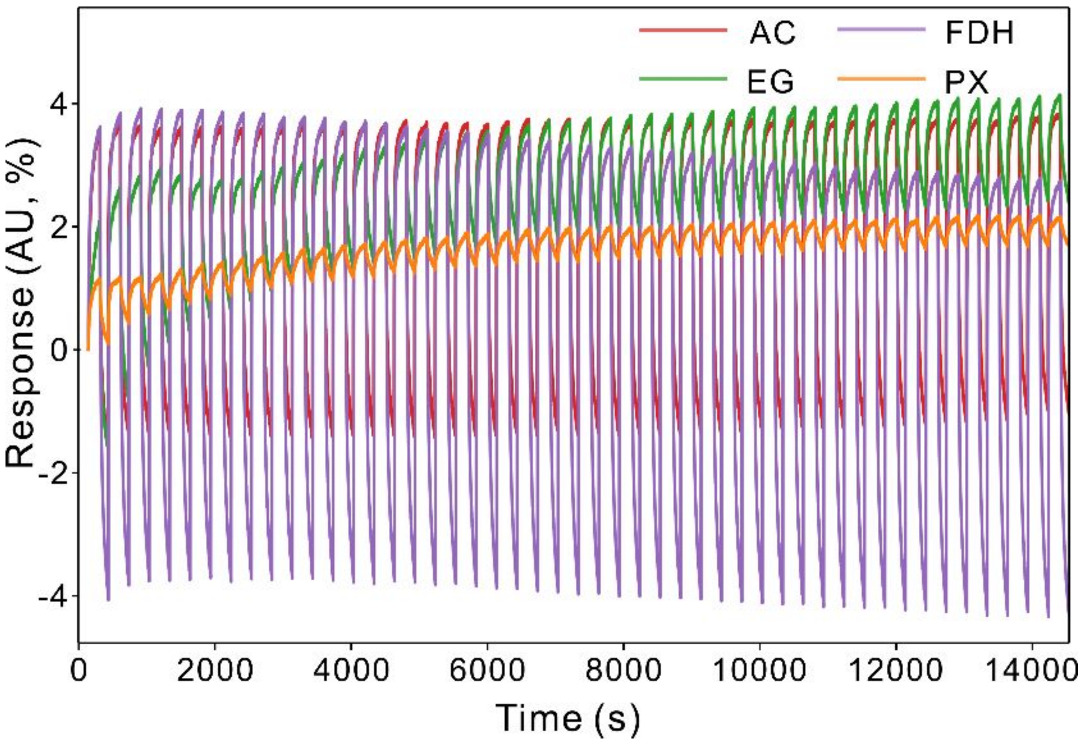

Figure S14. Typical sensing response profile of Cu-BHT NSs-based sensor towards analyte gas (PX, FDH, AC, and EG) upon cycling exposure measurement. A full measurement is composed of 48 repetitions test for each analyte gas.

The sensing response *S* is calculates following the below formula:

$$S\,(\%) = \frac{\triangle R(t)}{R(0)} * 100 = \frac{I(0) - I(t)}{I(t)} * 100$$

where $\triangle$R is the resistance difference before analyte gas exposure and during analyte gas exposure, t is the time, R(0) and I(0) are the resistance and current before exposure (t=0), respectively. I(t) is the current monitored during the exposure period.

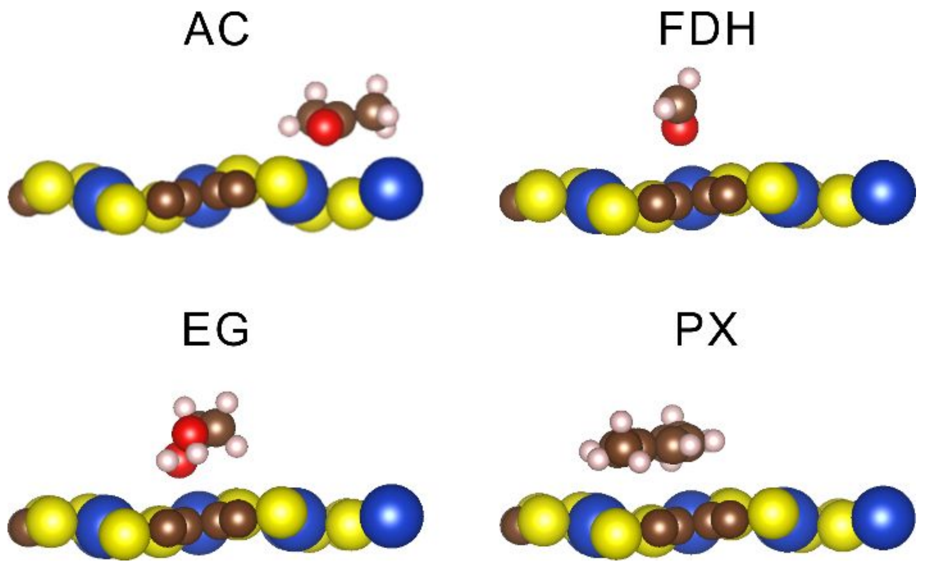

Figure S15. Snapshots of analyte gas molecules' most stable binding configuration on Cu-BHT NSs surface.

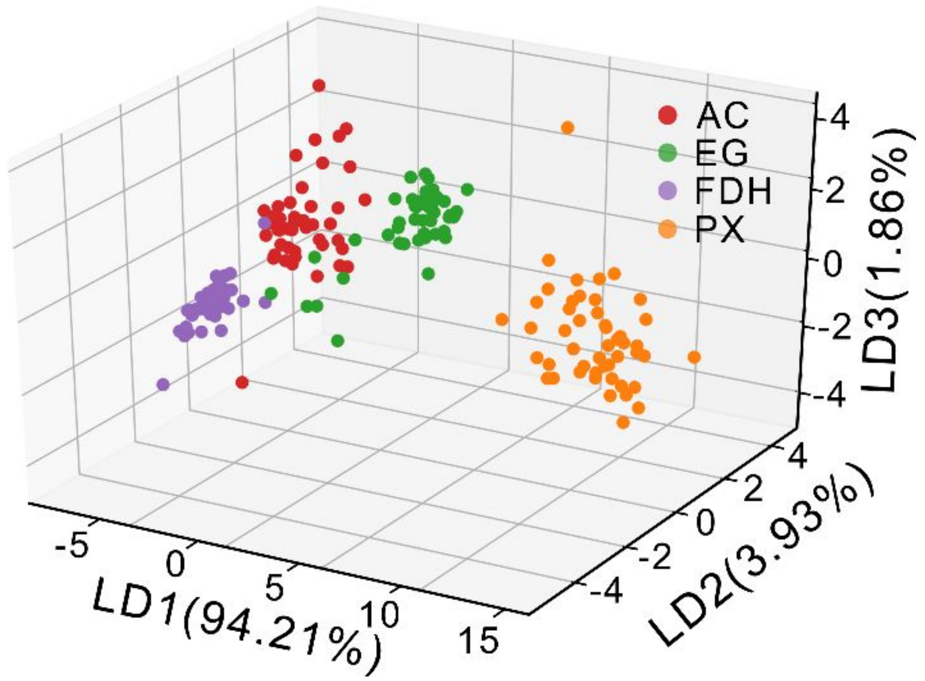

Figure S16. Linear discriminant analysis (LDA) score plot for FDH, AC, EG and PX analyte gases in 3D space.

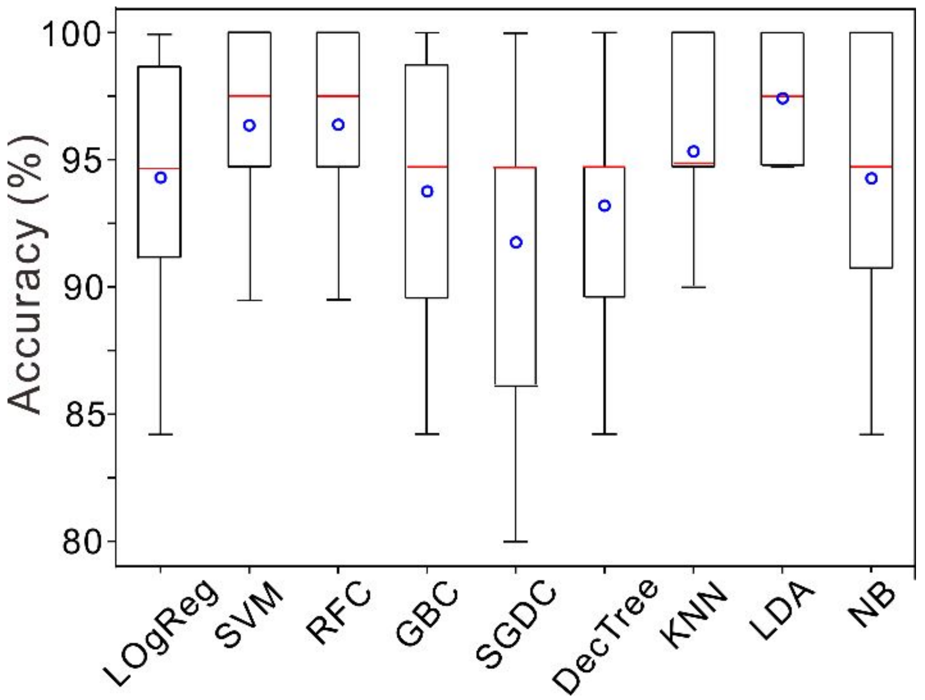

Figure S17. The prediction accuracy of developed nanosensors relationship with classifier algorithms using k-fold cross-validation approach (k=10).

Table S1. The electrical conductivity of Cu-BHT plates was measured at room temperature. Conductivity of the MOF measured by four-point probe method.

2D *c*-MOFs samples	Conductivity (S cm^{-1})
Cu-BHT NSs	0.79
Bulk Cu-BHT NPs	91.32
Cu-HHTP NSs	9.57E-04
Cu-HHB NSs	5.41E-06
Co-HHTP NSs	7.71E-05
Zn-HHTP NSs	1.21E-05

Table S2. Comparison of conductive MOF NSs by different synthesis method.

Method	MOF NSs	Thickness (nm)	Lateral size	Scalability	Ref.
Surfactant-Assisted Synthesis	HHB-Cu NSs	4.2 ± 1.1	0.30-0.65 μm^2	yes	1
	HHB-Ni NSs	4.5 ± 1.4	0.25-0.56 μm^2	yes	
	HHTP-Cu NSs	5.1 ± 2.6	0.002-0.02 μm^2	yes	
Ball milling mechanical exfoliation	$Ni_2[CuPc(NH)_8]$ NSs	7	0.08 μm^2	yes	2
Spin-coating interfacial self-assembly approach	Cu-BHT NSs	5-35	centimeter-sized	yes	3
MOF nanosheet sacrifice approach	Cu-BHT NSs	5-25	0.25-16 μm^2	yes	This work

Table S3. The physical properties of analyte gases and their calculated concentration in this work.

ID.	Analyte gas	Structure	Formula	M	P	C
**1**	P-Xylene (PX)	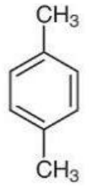	C_8H_{10}	106	0.900	1300
**3**	Formaldehyde (FDH) 37% aqueous solution	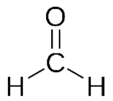	CH_2O	30	0.113	170
**4**	Acetone (AC)	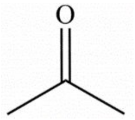	C_3H_6O	58	24.67	35500
**5**	Ethylene glycol (EG)	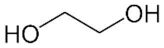	$C_2H_6O_2$	62	0.008	12

Note:
M: Mass (unit: g/mol);
P: Vapor pressure (unit: kPa) @ 20 °C;
C: Concentration (ppm) calculated according to the below equation.

Odor concentration could be determined *via* the below equation: [8-9]

$$C(ppm) = 10^6 \times \frac{P_{gas}}{P_0} \times \frac{v_c}{v_c + v_d} \qquad \text{Equation 1}$$

Where, P_{gas} is the saturated vapor pressure of analyte gas at room temperature as indicated in above table, P_0 is the input nitrogen gas pressure (760 mmHg), V_c and V_d are the flow rate (sccm) of both carrier gas (300 sccm) and dilution gas (1700 sccm) in this work, respectively. In our case, atmospheric pressure is applied and the working temperature is room temperature.

References

(1) Wang, Z.; Wang, G.; Qi, H.; Wang, M.; Wang, M.; Park, S.; Wang, H.; Yu, M.; Kaiser, U.; Fery, A., Ultrathin two-dimensional conjugated metal–organic framework single-crystalline nanosheets enabled by surfactant-assisted synthesis. *Chem. Sci.* **2020,** *11* (29), 7665-7671.

(2) Wang, M.; Shi, H.; Zhang, P.; Liao, Z.; Wang, M.; Zhong, H.; Schwotzer, F.; Nia, A. S.; Zschech, E.; Zhou, S., Phthalocyanine-based 2D conjugated metal-organic framework nanosheets for high-performance micro-supercapacitors. *Adv. Funct. Mater.* **2020,** *30* (30), 2002664.

(3) Chen, X.; Lu, Y.; Dong, J.; Ma, L.; Yi, Z.; Wang, Y.; Wang, L.; Wang, S.; Zhao, Y.; Huang, J., Ultrafast in situ synthesis of large-area conductive metal–organic frameworks on substrates for flexible chemiresistive sensing. *ACS Appl. Mater. Interfaces* **2020,** *12* (51), 57235-57244.

(4) Nguyen, H.; El-Safty, S. A., Meso- and Macroporous Co_3O_4 Nanorods for Effective VOC Gas Sensors. *J. Phys. Chem. C* **2011,** *115* (17), 8466-8474.

(5) Yu, Y.; Wu, E.; Chen, Y.; Feng, Z.; Zheng, S.; Zhang, H.; Pang, W.; Liu, J.; Zhang, D., Volatile organic compounds discrimination based on dual mode detection. *Nanotechnology* **2018,** *29*, 245502.
